# *N*′-(2-Hydr­oxy-5-nitro­benzyl­idene)-2-(1*H*-indol-3-yl)acetohydrazide

**DOI:** 10.1107/S1600536808026044

**Published:** 2008-08-16

**Authors:** Subramaniam Puvaneswary, Hapipah M. Ali, Ward T. Robinson, Seik Weng Ng

**Affiliations:** a Department of Chemistry, University of Malaya, 50603 Kuala Lumpur, Malaysia

## Abstract

The mol­ecule of the title compound, C_17_H_14_N_4_O_4_, uses its amide –NH– group to form a hydrogen bond to the amido –C(=O)– group of an adjacent mol­ecule to furnish a linear chain structure. The hydr­oxy group forms an intra­molecular hydrogen bond; the indolyl –NH– unit does not engage in any strong hydrogen-bonding inter­actions.

## Related literature

For similar compounds, see: Martin Reyes *et al.* (1986 ▶); Martin Zarza *et al.* (1989 ▶).
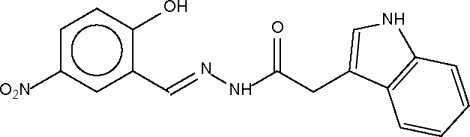


## Experimental

### 

#### Crystal data

C_17_H_14_N_4_O_4_
*M* *_r_* = 338.32
Orthorhombic, 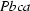

*a* = 9.5387 (2) Å
*b* = 11.2724 (3) Å
*c* = 29.7796 (7) Å
*V* = 3202.0 (1) Å^3^
*Z* = 8
Mo *K*α radiation
μ = 0.10 mm^−1^
*T* = 100 (2) K
0.30 × 0.25 × 0.20 mm

#### Data collection

Bruker SMART APEX diffractometer
Absorption correction: none
47721 measured reflections
3679 independent reflections
2059 reflections with *I* > 2σ(*I*)
*R*_int_ = 0.052

#### Refinement

*R*[*F* ^2^ > 2σ(*F* ^2^)] = 0.044
*wR*(*F* ^2^) = 0.160
*S* = 1.02
3679 reflections
228 parameters
H-atom parameters constrained
Δρ_max_ = 0.17 e Å^−3^
Δρ_min_ = −0.21 e Å^−3^

### 

Data collection: *APEX2* (Bruker, 2007 ▶); cell refinement: *SAINT* (Bruker, 2007 ▶); data reduction: *SAINT*; program(s) used to solve structure: *SHELXS97* (Sheldrick, 2008 ▶); program(s) used to refine structure: *SHELXL97* (Sheldrick, 2008 ▶); molecular graphics: *X-SEED* (Barbour, 2001 ▶); software used to prepare material for publication: *publCIF* (Westrip, 2008 ▶).

## Supplementary Material

Crystal structure: contains datablocks global, I. DOI: 10.1107/S1600536808026044/bq2091sup1.cif

Structure factors: contains datablocks I. DOI: 10.1107/S1600536808026044/bq2091Isup2.hkl

Additional supplementary materials:  crystallographic information; 3D view; checkCIF report

## Figures and Tables

**Table 1:** Hydrogen-bond geometry (Å, °)

*D*—H⋯*A*	*D*—H	H⋯*A*	*D*⋯*A*	*D*—H⋯*A*
O1—H1*o*⋯N2	0.84	1.85	2.583 (2)	146
N3—H3*n*⋯O4^i^	0.88	2.07	2.827 (2)	144

Symmetry code: (i) 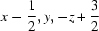.

**Fig. 1.**
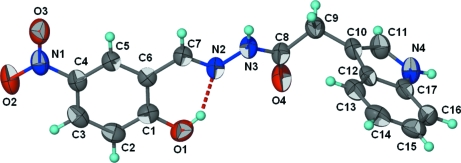
Thermal ellipsoid plot of (I) (Barbour, 2001) at the 50% probability level. Dashed line indicates H-bonding.

## supplementary crystallographic information

### Comment

There are many examples of Schiff bases derived from the condensation of salicylaldehyde and substituted salicyldehydes with hydrazides such as the ones reported by Martin Reyes *et al.* (1986) and Martin Zarza *et al.* (1989). The title compound (Fig. 1) is another example. The molecule uses its amido –NH– group to form a hydrogen bond to the amido –C(=O)– group of an adjacent molecule to furnish a linear chain structure.

### Experimental

The Schiff base was prepared by refluxing a solution of indole-3-acetic acid hydrazide (0.34 g, 1.80 mmol) and 5-nitrosalicylaldehyde (0.30 g, 1.80 mmol) in acidified ethanol (25 ml) for 2 h. On cooling to room temperature, yellow crystals separated out.

### Refinement

All H-atoms were placed in calculated positions (C—H 0.95, N—H 0.88, O–H 0.84 Å) and were included in the refinement in the riding model approximation, with *U*(H) set to 1.2 to 1.5*U*_eq_(C,*N*,*O*).

### Crystal data

**Table:**

C_17_H_14_N_4_O_4_	*F*_000_ = 1408
*M**_r_* = 338.32	*D*_x_ = 1.404 Mg m^−^^3^
Orthorhombic, *P**b**c**a*	Mo *K*α radiation λ = 0.71073 Å
Hall symbol: -P 2ac 2ab	Cell parameters from 3679 reflections
*a* = 9.5387 (2) Å	θ = 2.5–22.2º
*b* = 11.2724 (3) Å	µ = 0.10 mm^−^^1^
*c* = 29.7796 (7) Å	*T* = 100 (2) K
*V* = 3202.0 (1) Å^3^	Irregular block, yellow
*Z* = 8	0.30 × 0.25 × 0.20 mm

### Data collection

**Table:**

Bruker SMART APEX diffractometer	2059 reflections with *I* > 2σ(*I*)
Radiation source: fine-focus sealed tube	*R*_int_ = 0.053
Monochromator: graphite	θ_max_ = 27.5º
*T* = 100(2) K	θ_min_ = 1.4º
ω scans	*h* = −12→12
Absorption correction: None	*k* = −14→13
47721 measured reflections	*l* = −38→38
3679 independent reflections	

### Refinement

**Table:**

Refinement on *F*^2^	Hydrogen site location: inferred from neighbouring sites
Least-squares matrix: full	H-atom parameters constrained
*R*[*F*^2^ > 2σ(*F*^2^)] = 0.044	*w* = 1/[σ^2^(*F*_o_^2^) + (0.0885*P*)^2^] where *P* = (*F*_o_^2^ + 2*F*_c_^2^)/3
*wR*(*F*^2^) = 0.160	(Δ/σ)_max_ = 0.001
*S* = 1.02	Δρ_max_ = 0.18 e Å^−^^3^
3679 reflections	Δρ_min_ = −0.21 e Å^−^^3^
228 parameters	Extinction correction: SHELXL97 (Sheldrick, 2008), Fc^*^=kFc[1+0.001xFc^2^λ^3^/sin(2θ)]^-1/4^
Primary atom site location: structure-invariant direct methods	Extinction coefficient: 0.007 (1)
Secondary atom site location: difference Fourier map	

### Special details

**Table:**

Geometry. All e.s.d.'s (except the e.s.d. in the dihedral angle between two l.s. planes) are estimated using the full covariance matrix. The cell e.s.d.'s are taken into account individually in the estimation of e.s.d.'s in distances, angles and torsion angles; correlations between e.s.d.'s in cell parameters are only used when they are defined by crystal symmetry. An approximate (isotropic) treatment of cell e.s.d.'s is used for estimating e.s.d.'s involving l.s. planes.

### Fractional atomic coordinates and isotropic or equivalent isotropic displacement parameters (Å^2^)

**Table:**

	*x*	*y*	*z*	*U*_iso_*/*U*_eq_	
O1	0.76143 (16)	0.34962 (15)	0.66810 (5)	0.0747 (5)	
H1O	0.7344	0.4058	0.6845	0.112*	
O2	0.50825 (18)	0.27110 (16)	0.47819 (5)	0.0896 (6)	
O3	0.36688 (19)	0.40496 (16)	0.50246 (5)	0.0853 (5)	
O4	0.78329 (15)	0.61201 (15)	0.75230 (5)	0.0790 (5)	
N1	0.4670 (2)	0.33901 (17)	0.50757 (6)	0.0647 (5)	
N2	0.60812 (15)	0.52866 (14)	0.69094 (5)	0.0522 (4)	
N3	0.56843 (16)	0.61209 (15)	0.72184 (5)	0.0543 (5)	
H3N	0.4821	0.6397	0.7223	0.065*	
N4	0.79603 (19)	0.75966 (17)	0.89103 (6)	0.0712 (5)	
H4N	0.8598	0.7879	0.9095	0.085*	
C1	0.6858 (2)	0.34823 (18)	0.63004 (7)	0.0563 (5)	
C2	0.7229 (2)	0.26733 (18)	0.59698 (8)	0.0651 (6)	
H2	0.7980	0.2137	0.6022	0.078*	
C3	0.6523 (2)	0.26386 (18)	0.55689 (7)	0.0625 (6)	
H3	0.6787	0.2090	0.5342	0.075*	
C4	0.5426 (2)	0.34134 (17)	0.55005 (6)	0.0538 (5)	
C5	0.5011 (2)	0.42048 (16)	0.58253 (6)	0.0511 (5)	
H5	0.4237	0.4715	0.5772	0.061*	
C6	0.57211 (19)	0.42584 (16)	0.62300 (6)	0.0473 (5)	
C7	0.53086 (19)	0.51242 (17)	0.65658 (6)	0.0505 (5)	
H7	0.4468	0.5567	0.6530	0.061*	
C8	0.6644 (2)	0.65087 (18)	0.75150 (6)	0.0555 (5)	
C9	0.6133 (2)	0.7485 (2)	0.78219 (6)	0.0643 (6)	
H9A	0.6427	0.8262	0.7699	0.077*	
H9B	0.5095	0.7472	0.7832	0.077*	
C10	0.6695 (2)	0.73543 (17)	0.82885 (6)	0.0546 (5)	
C11	0.7735 (2)	0.7979 (2)	0.84821 (7)	0.0684 (6)	
H11	0.8242	0.8600	0.8340	0.082*	
C12	0.62245 (19)	0.65262 (16)	0.86168 (7)	0.0514 (5)	
C13	0.5173 (2)	0.56695 (18)	0.86298 (8)	0.0626 (6)	
H13	0.4599	0.5530	0.8374	0.075*	
C14	0.4978 (3)	0.50326 (19)	0.90151 (9)	0.0745 (7)	
H14	0.4262	0.4447	0.9024	0.089*	
C15	0.5803 (3)	0.5221 (2)	0.93952 (8)	0.0753 (7)	
H15	0.5640	0.4760	0.9657	0.090*	
C16	0.6847 (2)	0.6062 (2)	0.93974 (7)	0.0667 (6)	
H16	0.7411	0.6194	0.9655	0.080*	
C17	0.7037 (2)	0.67047 (18)	0.90073 (7)	0.0561 (5)	

### Atomic displacement parameters (Å^2^)

**Table:**

	*U*^11^	*U*^22^	*U*^33^	*U*^12^	*U*^13^	*U*^23^
O1	0.0634 (10)	0.0901 (11)	0.0707 (10)	0.0201 (8)	−0.0089 (8)	0.0030 (8)
O2	0.0991 (14)	0.1023 (13)	0.0672 (11)	−0.0122 (10)	0.0139 (9)	−0.0333 (10)
O3	0.0835 (12)	0.0954 (12)	0.0771 (11)	0.0045 (10)	−0.0245 (9)	−0.0215 (9)
O4	0.0393 (9)	0.1232 (13)	0.0745 (11)	0.0156 (8)	−0.0106 (7)	−0.0360 (9)
N1	0.0663 (12)	0.0712 (12)	0.0566 (11)	−0.0201 (10)	0.0062 (9)	−0.0134 (10)
N2	0.0406 (9)	0.0683 (10)	0.0477 (9)	−0.0033 (8)	0.0012 (7)	−0.0058 (8)
N3	0.0360 (8)	0.0762 (11)	0.0507 (10)	0.0042 (8)	0.0002 (7)	−0.0134 (8)
N4	0.0664 (12)	0.0860 (13)	0.0612 (11)	−0.0182 (10)	−0.0085 (9)	−0.0171 (10)
C1	0.0460 (11)	0.0607 (12)	0.0623 (13)	−0.0011 (9)	0.0046 (10)	0.0044 (10)
C2	0.0548 (13)	0.0590 (13)	0.0814 (16)	0.0065 (10)	0.0103 (12)	0.0004 (11)
C3	0.0592 (14)	0.0557 (12)	0.0724 (15)	−0.0079 (10)	0.0201 (12)	−0.0112 (10)
C4	0.0512 (12)	0.0533 (11)	0.0568 (12)	−0.0139 (9)	0.0058 (10)	−0.0048 (9)
C5	0.0460 (11)	0.0536 (11)	0.0536 (11)	−0.0039 (9)	0.0030 (8)	−0.0029 (9)
C6	0.0409 (10)	0.0498 (10)	0.0513 (11)	−0.0052 (8)	0.0050 (8)	0.0011 (9)
C7	0.0416 (11)	0.0583 (11)	0.0515 (11)	−0.0014 (9)	0.0018 (9)	−0.0006 (9)
C8	0.0428 (12)	0.0750 (13)	0.0488 (11)	0.0012 (10)	−0.0009 (9)	−0.0068 (10)
C9	0.0573 (13)	0.0742 (14)	0.0613 (13)	0.0078 (11)	−0.0020 (10)	−0.0119 (11)
C10	0.0496 (12)	0.0617 (12)	0.0524 (11)	−0.0006 (9)	0.0004 (9)	−0.0154 (9)
C11	0.0671 (15)	0.0730 (14)	0.0650 (14)	−0.0150 (12)	0.0039 (11)	−0.0092 (11)
C12	0.0443 (11)	0.0532 (11)	0.0568 (12)	0.0047 (9)	0.0009 (9)	−0.0189 (9)
C13	0.0534 (13)	0.0564 (12)	0.0781 (15)	−0.0004 (10)	−0.0012 (11)	−0.0154 (11)
C14	0.0665 (15)	0.0541 (12)	0.103 (2)	−0.0025 (11)	0.0122 (14)	−0.0064 (13)
C15	0.0858 (18)	0.0594 (13)	0.0808 (17)	0.0180 (13)	0.0207 (14)	0.0039 (12)
C16	0.0717 (15)	0.0718 (14)	0.0566 (13)	0.0192 (13)	−0.0007 (11)	−0.0090 (11)
C17	0.0523 (12)	0.0578 (12)	0.0583 (12)	0.0059 (10)	0.0013 (10)	−0.0171 (10)

### Geometric parameters (Å, °)

**Table:**

O1—C1	1.344 (2)	C5—H5	0.9500
O1—H1O	0.8400	C6—C7	1.452 (3)
O2—N1	1.227 (2)	C7—H7	0.9500
O3—N1	1.220 (2)	C8—C9	1.512 (3)
O4—C8	1.216 (2)	C9—C10	1.497 (3)
N1—C4	1.456 (3)	C9—H9A	0.9900
N2—C7	1.274 (2)	C9—H9B	0.9900
N2—N3	1.369 (2)	C10—C11	1.347 (3)
N3—C8	1.345 (2)	C10—C12	1.424 (3)
N3—H3N	0.8800	C11—H11	0.9500
N4—C11	1.363 (3)	C12—C13	1.393 (3)
N4—C17	1.367 (3)	C12—C17	1.412 (3)
N4—H4N	0.8800	C13—C14	1.366 (3)
C1—C2	1.388 (3)	C13—H13	0.9500
C1—C6	1.409 (3)	C14—C15	1.395 (3)
C2—C3	1.371 (3)	C14—H14	0.9500
C2—H2	0.9500	C15—C16	1.375 (3)
C3—C4	1.378 (3)	C15—H15	0.9500
C3—H3	0.9500	C16—C17	1.381 (3)
C4—C5	1.374 (3)	C16—H16	0.9500
C5—C6	1.384 (3)		
			
C1—O1—H1O	109.5	O4—C8—C9	123.48 (18)
O3—N1—O2	122.85 (19)	N3—C8—C9	114.46 (18)
O3—N1—C4	119.01 (18)	C10—C9—C8	111.95 (17)
O2—N1—C4	118.1 (2)	C10—C9—H9A	109.2
C7—N2—N3	118.59 (16)	C8—C9—H9A	109.2
C8—N3—N2	118.45 (16)	C10—C9—H9B	109.2
C8—N3—H3N	120.8	C8—C9—H9B	109.2
N2—N3—H3N	120.8	H9A—C9—H9B	107.9
C11—N4—C17	109.21 (17)	C11—C10—C12	106.33 (18)
C11—N4—H4N	125.4	C11—C10—C9	127.6 (2)
C17—N4—H4N	125.4	C12—C10—C9	126.11 (18)
O1—C1—C2	117.97 (19)	C10—C11—N4	110.5 (2)
O1—C1—C6	122.12 (18)	C10—C11—H11	124.7
C2—C1—C6	119.91 (19)	N4—C11—H11	124.7
C3—C2—C1	120.8 (2)	C13—C12—C17	118.12 (19)
C3—C2—H2	119.6	C13—C12—C10	134.45 (19)
C1—C2—H2	119.6	C17—C12—C10	107.40 (17)
C2—C3—C4	118.89 (19)	C14—C13—C12	119.1 (2)
C2—C3—H3	120.6	C14—C13—H13	120.5
C4—C3—H3	120.6	C12—C13—H13	120.5
C5—C4—C3	121.73 (19)	C13—C14—C15	121.7 (2)
C5—C4—N1	118.73 (19)	C13—C14—H14	119.2
C3—C4—N1	119.54 (18)	C15—C14—H14	119.2
C4—C5—C6	120.04 (18)	C16—C15—C14	121.2 (2)
C4—C5—H5	120.0	C16—C15—H15	119.4
C6—C5—H5	120.0	C14—C15—H15	119.4
C5—C6—C1	118.63 (17)	C15—C16—C17	116.9 (2)
C5—C6—C7	119.78 (17)	C15—C16—H16	121.5
C1—C6—C7	121.58 (18)	C17—C16—H16	121.5
N2—C7—C6	119.53 (17)	N4—C17—C16	130.4 (2)
N2—C7—H7	120.2	N4—C17—C12	106.52 (18)
C6—C7—H7	120.2	C16—C17—C12	123.1 (2)
O4—C8—N3	122.03 (18)		
			
C7—N2—N3—C8	−163.35 (18)	N3—C8—C9—C10	142.14 (19)
O1—C1—C2—C3	−178.10 (19)	C8—C9—C10—C11	103.7 (2)
C6—C1—C2—C3	1.7 (3)	C8—C9—C10—C12	−76.0 (3)
C1—C2—C3—C4	−0.7 (3)	C12—C10—C11—N4	0.4 (2)
C2—C3—C4—C5	−0.9 (3)	C9—C10—C11—N4	−179.39 (19)
C2—C3—C4—N1	179.78 (17)	C17—N4—C11—C10	−0.8 (2)
O3—N1—C4—C5	−1.9 (3)	C11—C10—C12—C13	177.9 (2)
O2—N1—C4—C5	177.32 (17)	C9—C10—C12—C13	−2.4 (3)
O3—N1—C4—C3	177.38 (18)	C11—C10—C12—C17	0.1 (2)
O2—N1—C4—C3	−3.4 (3)	C9—C10—C12—C17	179.90 (18)
C3—C4—C5—C6	1.6 (3)	C17—C12—C13—C14	−0.6 (3)
N1—C4—C5—C6	−179.14 (16)	C10—C12—C13—C14	−178.1 (2)
C4—C5—C6—C1	−0.5 (3)	C12—C13—C14—C15	0.1 (3)
C4—C5—C6—C7	177.88 (16)	C13—C14—C15—C16	0.2 (3)
O1—C1—C6—C5	178.74 (17)	C14—C15—C16—C17	−0.1 (3)
C2—C1—C6—C5	−1.0 (3)	C11—N4—C17—C16	−178.7 (2)
O1—C1—C6—C7	0.3 (3)	C11—N4—C17—C12	0.8 (2)
C2—C1—C6—C7	−179.43 (17)	C15—C16—C17—N4	179.0 (2)
N3—N2—C7—C6	178.97 (15)	C15—C16—C17—C12	−0.4 (3)
C5—C6—C7—N2	−170.09 (17)	C13—C12—C17—N4	−178.75 (16)
C1—C6—C7—N2	8.3 (3)	C10—C12—C17—N4	−0.6 (2)
N2—N3—C8—O4	−1.8 (3)	C13—C12—C17—C16	0.8 (3)
N2—N3—C8—C9	176.17 (17)	C10—C12—C17—C16	178.93 (18)
O4—C8—C9—C10	−39.9 (3)		

### Hydrogen-bond geometry (Å, °)

**Table:**

*D*—H···*A*	*D*—H	H···*A*	*D*···*A*	*D*—H···*A*
O1—H1o···N2	0.84	1.85	2.583 (2)	146
N3—H3n···O4^i^	0.88	2.07	2.827 (2)	144
N4—H4n···O2^ii^	0.88	2.49	3.216 (2)	140

Symmetry codes: (i) *x*−1/2, *y*, −*z*+3/2; (ii) −*x*+3/2, −*y*+1, *z*+1/2.
